# Responses to pathogen exposure in sentinel juvenile fall-run Chinook salmon in the Sacramento River, CA

**DOI:** 10.1093/conphys/coad066

**Published:** 2023-08-28

**Authors:** Samah M R Abdelrazek, Richard E Connon, Camilo Sanchez, Benjamin Atencio, Florian Mauduit, Brendan Lehman, Sascha L Hallett, Stephen D Atkinson, J Scott Foott, Miles E Daniels

**Affiliations:** Department of Anatomy, Physiology and Cell Biology, University of California, Davis, Davis, CA 95616, USA; Department of Anatomy, Physiology and Cell Biology, University of California, Davis, Davis, CA 95616, USA; Department of Anatomy, Physiology and Cell Biology, University of California, Davis, Davis, CA 95616, USA; Institute of Marine Sciences, University of California, Santa Cruz, Affiliated with Southwest Fisheries Science Center, National Marine Fisheries Service, National Oceanic and Atmospheric Administration, Santa Cruz, CA 95060, USA; Department of Anatomy, Physiology and Cell Biology, University of California, Davis, Davis, CA 95616, USA; Institute of Marine Sciences, University of California, Santa Cruz, Affiliated with Southwest Fisheries Science Center, National Marine Fisheries Service, National Oceanic and Atmospheric Administration, Santa Cruz, CA 95060, USA; Department of Microbiology, Oregon State University, Corvallis, OR 97331, USA; Department of Microbiology, Oregon State University, Corvallis, OR 97331, USA; California Nevada Fish Health Center, U.S. Fish and Wildlife Service, Anderson, CA 96007, USA; Institute of Marine Sciences, University of California, Santa Cruz, Affiliated with Southwest Fisheries Science Center, National Marine Fisheries Service, National Oceanic and Atmospheric Administration, Santa Cruz, CA 95060, USA

**Keywords:** Chinook salmon, gene expression, pathogens

## Abstract

This study investigated how the deployment of juvenile Chinook salmon in ambient river conditions and the subsequent exposure to and infection by pathogens was associated with the changes in the expression of genes involved in immune system functioning, general stress and host development. Juvenile fish were deployed in sentinel cages for 21 days in the Sacramento River, CA, USA. Gill, kidney and intestinal tissue were sampled at 0, 7, 14 and 21 days post-deployment. Pathogen detection and host response were assessed by a combination of molecular and histopathological evaluation. Our findings showed that fish became infected by the parasites *Ceratonova shasta*, *Parvicapsula minibicornis* and *Ichthyophthirius multifiliis,* and to a lesser extent, the bacteria *Flavobacterium columnare* and *Rickettsia*-like organisms. Co-infection was common among sentinel fish. Expression of investigated genes was altered following deployment and was often associated with pathogen abundance. This study provides a foundation for future avenues of research investigating pathogens that affect out-migrating Chinook salmon in the Sacramento River, and offers crucial knowledge related to conservation efforts.

## Teaser

Sentinel juvenile Chinook salmon deployed in the Sacramento River were co-infected with Ceratonova shasta, Parvicapsula minibicornis, and Ichthyophthirius multifiliis. Upregulation of immune genes was associated with pathogen abundance in gill and kidney tissues. Infection and gene response was observed as early at 7 days post deployment.

## Introduction

Exposure to pathogens (disease-causing organisms) can play an important role in salmon ecology and population health (Johnson *et al.*, 2015; Sofonea *et al.*, 2017; Chapman *et al.*, 2021; Teffer *et al.*, 2022). This role can also be dynamic as rapid environmental changes disrupt the host–pathogen relationships for salmon, affecting pathogen species composition, host immune responses and overall animal performance (Altizer *et al.*, 2011; Evans *et al.*, 2011). For example, while many opportunistic pathogens may be endemic in a watershed, diseases may only manifest when other factors, such as poor environmental conditions, first compromise the salmon host’s ability to fight infection (Miller *et al.*, 2014).

The epidemiological triad of host–environment–agent is the core concept used to describe how these three factors interact and determine disease dynamics (Dicker *et al.*, 2006). Research on these interactions, however, has often focused on a single pathogen at a time or only looked at a single point in time. Many times, salmon are exposed to multiple pathogens, and these multiple exposures may synergize and not result in simple additive effects on the host. While it is suspected that co-infection from multiple pathogens has important consequences for host response (Sofonea *et al.*, 2015; Sofonea *et al.*, 2017), predicting outcomes of multiple exposures remains challenging.

The health condition of a salmon prior to exposure relates to its ability to mount an immune response to prevent, control and eliminate pathogens. For many fish species, the gill serves as a primary entryway for pathogens (Teffer and Miller, 2019); however, the mucosal immune system of a healthy fish also provides protection against pathogens via an array of interacting innate and adaptive immune cells and molecules (Gomez *et al.*, 2013). Therefore, gene expression, as measured from salmon gill biopsies, can indicate whether a fish is responding physiologically to pathogen infections and the magnitude of that response (Connon *et al.*, 2012; Jeffries *et al.*, 2014; Teffer *et al.*, 2017). However, gills being an external organ are not reliable indicators of systemic infections. In fish, the kidney is a major lympho-haematopoietic organ (Zapata *et al.*, 2006) and is routinely sampled in fisheries pathology procedures with the intent to determine the extent of systemic infection (Bjørgen and Koppang, 2022).

Pacific salmon populations have declined across their ranges due to overexploitation, habitat loss, reduced prey availability and pathogens (Rand, 2002; Ruckelshaus *et al.*, 2002; Kent, 2011; Bass *et al.*, 2022). Central Valley fall-run Chinook salmon (*Oncorhynchus tshawytscha*), the focus of this study, has been designated as a ‘species of concern’ by National Oceanic and Atmospheric Administration (NOAA), necessitating extensive research to quantify and understand factors threatening their survival (Buchanan *et al.*, 2018). During out-migration, Central Valley fall-run Chinook salmon may be exposed to a range of pathogens that can affect growth, development, distribution, behaviour and predation risk for individual survival (Myrick and Cech 2001, 2004).

Two salmon pathogens of particular concern in the Sacramento River, which are often detected at a juvenile fish monitoring station near the Red Bluff Diversion Dam (RBDD) (Foott *et al.*, 2017) and elsewhere (Mauduit *et al.*, 2022), are the myxozoan parasites *Ceratonova shasta* and *Parvicapsula minibicornis*. *Ceratonova shasta* invades salmon via the gill, then migrates to the target tissue (Bjork and Bartholomew, 2010); how *P. minibicornis* enters salmon is unknown. *Ceratonova shasta* primarily infects the salmon intestinal tract, but can also infect other tissues, while *P. minibicornis* primarily infects the kidney. Research comparing the abundance of *C. shasta*, *P. minibicornis* and gene expression profiles from gill biopsies following 14-day exposures in the Sacramento River further found that the copy number of pathogens were correlated with a significant upregulation of a proinflammatory response gene (SAA, Mauduit *et al.*, 2022). However, Mauduit *et al.* (2022) only focused on two pathogens detected in gill tissue during a 14-day exposure, and there has been limited research related to other pathogens known to exist in the system (Lehman *et al.*, 2020).

The goals of this study were to build upon the findings from prior research in the Sacramento River and further characterize pathogen infections and gene regulation in sentinel juvenile Chinook salmon. The specific objectives were to (1) assess weekly changes in the prevalence and abundance of pathogens in the tissue of fish over the course of a 21-day exposure to river conditions at the RBDD site, (2) characterize patterns in gene regulation in both gill and kidney tissues over this same time and (3) identify associations between measured levels of pathogen exposure and expression of immune, stress and development-related genes to develop a clearer picture of salmon health.

## Materials and Methods

### Sentinel fish exposure

All fish care and protocols were reviewed and approved by the University of California, Santa Cruz Institutional Animal Care and Use Committee, protocol no. Danim1911. We used fall-run Chinook that were spawned at Coleman National Fish Hatchery (CNFH; near Anderson, CA, USA) and raised at the NOAA Southwest Fisheries Science Center (SWFSC) in Santa Cruz, CA, USA. Juvenile fall-run Chinook salmon (mean fork length ± SD, 7.5 ± 0.5 cm; mean mass ± SD, 4.6 ± 0.9 g) were transported to the deployment location in rotomolded coolers and monitored regularly to maintain ~13.6°C and dissolved oxygen >90% during transport.

Sentinel fish were deployed for 21 days in May 2020. We chose this period of the year, fish of this size and length of deployment to align with the life history of fall run in the Sacramento River. Fall-run Chinook salmon emerge from gravel from December to March and migrate to the ocean from March to July, at a size of approximately 8 cm (Fisher, 1994). While it is difficult to know how long fish are rearing near the RBDD, and while this likely varies as a function environmental conditions and fish origin (i.e. wild vs hatchery), data collected from acoustic tagging programmes (CalFishTrack; https://oceanview.pfeg.noaa.gov/CalFishTrack/) of hatchery fish have recorded residence time as long as 25 days, while the majority were observed to spend less than 7 days in this section of the river.

**Figure 1:**
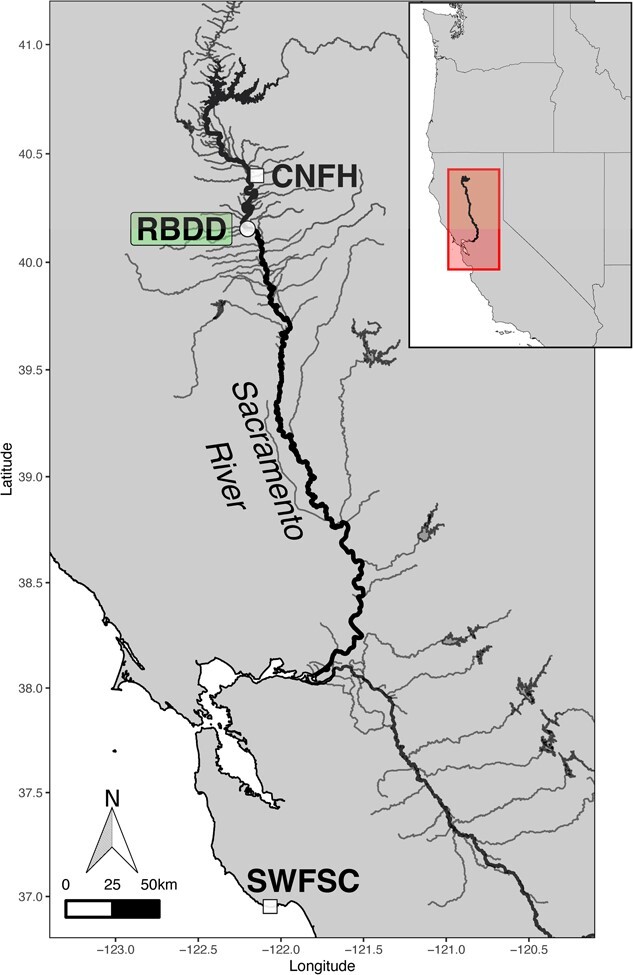
Map of the study area and fish facilities—CNFH, RBDD and the SWFSC.

Fish were held in cylindrical cages (diameter, 50 cm; height, 80 cm; *N* = 3 cages; 54 fish per cage) at the RBDD (Fig. 1). Cages were constructed with a welded steel rod skeleton, with 4-mm plastic drainage mesh covering the bottom and sides, and a 6-mm-mesh polyester netting covering the tops of cages, to allow food, such as drifting invertebrates, to enter and exit. All cages were suspended from the water surface using flotation devices and were fastened to the RBDD such that cages were not directly downstream from each other. Of the fish deployed, 95% were recovered, with the remainder lost and assumed to escape or die and decompose rapidly.

### Tissue collection

Seventy-five juvenile Chinook salmon were sampled for molecular analysis: Fish were sampled prior to deployment at the SWFSC facility and used as reference samples on Day 0 (*N* = 15), and at 7, 14 and 21 days post-deployment (dpd) at the RBDD site (*N* = 20; six to seven fish per cage). Immediately after euthanization, gill, kidney and intestinal tissue were collected and preserved in RNAlater before being transported at ambient temperature to the corresponding laboratory. Gill and kidney samples were stored at −80°C at the University of California, in Davis, on arrival, while the intestine samples were shipped to Oregon State University. On Day 21 (the endpoint of the study), an additional 20 fish that were separate from samples for molecular analysis were collected for histopathological assessments.

### Genomic DNA extraction, total RNA extraction and cDNA synthesis.

Prior to extraction, gill and kidney tissues were independently homogenized using a Qiagen TissueLyser LT (Qiagen, Valencia, CA, USA). A QIAcube system 230 V (Qiagen) was used to extract genomic DNA (gDNA), and total RNA from gill and kidney tissues using the DNeasy Blood and Tissue Kit (Qiagen) and RNeasy Plus Mini Kit (Qiagen), respectively, according to the manufacturer’s recommendations. Extracted gDNA and RNA were assessed for quality (A260:280 and A260:230 ratios) and concentration (ng μl^−1^) using a NanoDrop ND1000 spectrophotometer (NanoDrop Technologies, Inc., Wilmington, DE, USA). Integrity of RNA was assessed by electrophoresis on a 1% w/v agarose gel. A QuantiTect Reverse Transcription Kit (Qiagen) was used to synthesize cDNA from 1 μg of total RNA following the manufacturer’s protocol, with the exception that reaction volumes were scaled to a final volume of 40 μl. Fish intestine samples were digested using a modified ‘boiled-crude’ method of Palenzuela *et al.* (1999): incubation at 56°C for 1–2 h with 180-μl buffer ATL (Qiagen) and 20-μl proteinase K to digest tissue, followed by heat denaturation at 85°C for 15 min, then dilution 1:100 prior to amplification in polymerase chain reaction (PCR).

### Pathogen detection

Probe-based quantitative PCR (qPCR) was used for the detection and quantification of eight pathogens (four parasites and four bacteria) known to cause disease in salmon (Table 1). Custom TaqMan gene expression assays were designed for the gene expression portion of this study. Published TaqMan assays were sourced from Miller *et al.* (2016) and from Panangala *et al.* (2007). qPCR consisted of 6-μl TaqMan Environmental Master Mix 2.0 (Thermo Fisher Scientific), 0.9 μM each primer, 0.25 μM probe, 5-μl template gDNA and nuclease-free water to a total volume of 12 μl. Plasmids corresponding to each pathogen (Eurofins Genomics LLC, Louisville, KY, USA) were used to create standard curves for assay quality control and assurance. All DNA samples were amplified in 384-well plates in a QuantStudio7 Flex Real-Time PCR System (Real-time PCR Research and Diagnostics Core Facility, University of California, Davis), with 10-fold, serially diluted plasmid standards. All reactions were tested in triplicate, those with Ct ≤ 40 (fluorescence in at least two out of three wells) were considered positive; samples where two or more wells had an undetermined Ct (>40) were below the detection limit of the assay and interpreted as negative, under amplification conditions: 2 min at 50°C; 10 min at 95°C; followed by 40 cycles of 15 s at 95°C, and 1 min at 60°C. Fluorescent signals were analysed using QuantStudio Design and Analysis Software v2 (Thermo Fisher Scientific). While copy number per nanogram of gDNA is not directly comparable to pathogen abundance (except in the case of single-copy genes in a bacterium or virus; reviewed by Chapman *et al.*, 2021), the results for each pathogen in gill and kidney were represented by copy number per nanogram of gDNA, hereafter referred to as pathogen abundance. Extracted intestine gDNA was assayed solely for *C. shasta* ITS-1 rDNA region using conventional PCR described in Atkinson *et al.* (2018). Amplicons were visualized by gel electrophoresis, and PCR result was given as negative and positive.

**Table 1:** Pathogens screened for by quantitative polymerase chain reaction analyses in juvenile fall-run Chinook salmon (sourced from Miller *et al.*, 2016)

Pathogen Species	Agent type	Reference
*C. shasta*	Parasite	Hallett and Bartholomew (2006)
*P. minibicornis*	Parasite	Hallett and Bartholomew (2009)
*Myxobolus cerebralis*	Parasite	Kelley *et al.* (2004)
*I. multifiliis*	Parasite	Miller *et al*. (2016)
*Candidatus Branchiomonas cysticola*	Bacterium	Mitchell *et al.* (2013)
*F. columnare*	Bacterium	Panangala *et al.* (2007)
*Flavobacterium psychrophilum*	Bacterium	Duesund *et al.* (2010)
Rickettsia-like organisms	Bacterium	Lloyd *et al.* (2011)

### Gene expression

We examined the expression of 11 genes linked to stress, development and immunological responses, some of which have been the focus of recent pathogen research on salmonids (Bjork *et al.*, 2014; Jeffries *et al.*, 2014). Quantitative reverse transcription PCR was used to examine the gene expression profile in gill and kidney tissues (Table 2). Primers for each gene were either designed using Primer3 software from National Center for Biotechnology Information (NCBI) sequences or retrieved from published literature and synthesized by Integrated DNA Technologies (Coralville, IA, USA). Standard curves were generated using cDNA synthesized from pooled RNA. Each qPCR was in a final volume of 12 μl: 6 μl of 2X PowerUp SYBR Green Master Mix (Applied Biosystems, Foster City, CA, USA), 0.4 μM of the respective forward and reverse primers and 5 μl of cDNA, and RNase free water to volume. All measurements were performed in triplicate on 384-well plates using the QuantStudio7 Flex Real-Time PCR Systems together with QuantStudio Design and Analysis Software v2. The cycling conditions were 50°C for 2 min, 95°C for 2 min, followed by 40 cycles of 95°C for 15 s, 55°C for 15 s and 72°C for 1 min. A dissociation curve analysis was performed at 95°C for 15 s, 60°C for 1 min and 95°C for 15 s with each run to confirm specificity. Gene expression data were normalized to the mean of three reference genes, RPL7, Actβ and GAPDH, and presented relative to reference fish, sampled at Day 0. Quantitative cycle is reported as the geometric mean of assay triplicate, and relative expression of target genes was determined using the 2^−ΔΔCt^ method (Livak and Schmittgen, 2001) and is presented as log_2_ transformed fold change.

**Table 2:** Biomarkers of host immunity, stress and reference genes that were evaluated using quantitative polymerase chain reaction analyses in juvenile fall-run Chinook salmon

Gene	Abbreviation	Function	Reference
Tumour necrosis factor alpha	TNFα	Pro-inflammatory	Mauduit *et al.* (2022)
Chemokine Interleukin 1β	IL-1β	Pro-inflammatory	Mauduit *et al.* (2022)
Serum amyloid protein A	SAA	Pro-inflammatory	Mauduit *et al.* (2022)
Chemokine Interleukin 8	IL-8	Pro-inflammatory	Mauduit *et al.* (2022)
Chemokine Interleukin 6	IL-6	Pro-inflammatory	Bjork *et al.* (2014)
Interleukin 10	IL-10	Anti-inflammatory	Bjork *et al.* (2014)
Transforming growth factor β	TGFβ	Anti-inflammatory	Bjork *et al.* (2014)
Immunoglobulin T	IgT	Adaptive	This study^a^
Classical Immunoglobulin	IgM	Adaptive	Mauduit *et al.* (2022)
Heat Shock protein serpin H1	HSP47	General stress	This study^a^
Brain derived neurotropic factor	BDNF	Development	This study^a^
60S Ribosomal gene 7l	RPL7	Reference gene	Mauduit *et al.* (2022)
β-Actin	Actβ	Reference gene	Bjork *et al.* (2014)
Glyceraldehyde-3-phosphate dehydrogenase	GAPDH	Reference gene	This study^a^

a ^a^Primer sequences developed in-house. See Supplementary Table S1 for details.

### Histopathology

At 21 dpd, one-gill filament, posterior kidney tissue and 3–6 mm of the distal small intestine were fixed in Davidson’s fixative for 48 h, then stored in 70% ethanol for 24 h before being processed into 5-μm paraffin sections and stained with haematoxylin and eosin (Humason, 1979). Each resulting slide was examined blindly, at both low (×40) and high magnification (×400), and each fish received a score based on an initial tissue examination. Histological ratings for *C. shasta* in intestinal tissue and *P. minibicornis* in kidney tissue, the parasites’ primary infection sites, were graded on a scale of 0 to 2, with 0 = no parasites, 1 = parasite present in the respective tissue but with minimal inflammatory changes and 2 = multifocal lesions associated with the parasite infection. *Ceratonova shasta* 2 rating of intestine refers to lamina propria hyperplasia, necrotic epithelium/sloughing and necrotic muscularis. *Parvicapsula minibicornis* 2 rating of kidney refers to interstitial hyperplasia, necrotic interstitium or tubule, interstitial granuloma, glomerulonephritis and protein casts within the glomeruli or tubules.

### Statistical analysis

We used a non-parametric Kruskal-Wallis to test whether pathogen abundance changed in sentinel fish over the course of the study (i.e. from 0, 7, 14 and 21 dpd) (Kruskal and Wallis, 1952). We applied a significance threshold of *alpha* = 0.05 after adjusting for multi-hypothesis testing using a false discovery rate as outlined by Benjamini and Hochberg (1995). Pathogen abundance data were analysed as log_10_ transformed values to detect changes that occurred over an order of magnitude.

To test for associations between pathogen abundance in tissues and gene expression, we used linear regression and model selection based on Akaike information criterion (AIC) (Burnham *et al.*, 2010). Specifically, we hypothesized gene expression would be associated with pathogen abundance, exposure duration or both pathogen abundance and exposure duration. For each gene and each tissue (i.e. gill and kidney), we built four candidate models, where the outcome of interest in all models was expression of a particular gene, represented as log_2_ transformed fold change so that we could evaluate changes in gene expression that doubled. Model 1 (the global model) included the covariates of pathogen abundance and exposure duration and the interaction of the two as we assumed the effect of one may be dependent on the other. Model 2 only included the covariate pathogen abundance, and Model 3 only included the covariate exposure duration. Model 4 (the null model) assumed no relation to pathogen abundance or exposure duration. To select the most parsimonious model describing the relationship between gene expression and covariates, we used AIC corrected for small samples size (AICc) (Burnham *et al.*, 2010).

As we observed multiple pathogens in each tissue but wanted to constrain the number of regression analyses conducted, we calculated a summary pathogen abundance metric by summing abundance (i.e. copy numbers) for all pathogen species detected in each sample. This cumulative pathogen abundance covariate was analysed as log_10_ transformed values. Exposure duration was represented as 0, 7, 14 and 21 dpd. For interpretation, only the candidate model with the lowest AICc score was used. All analyses were performed in R (version 4.3.1; R-CoreTeam 2023).

## Results

### Pathogen prevalence and abundance

**Figure 2:**
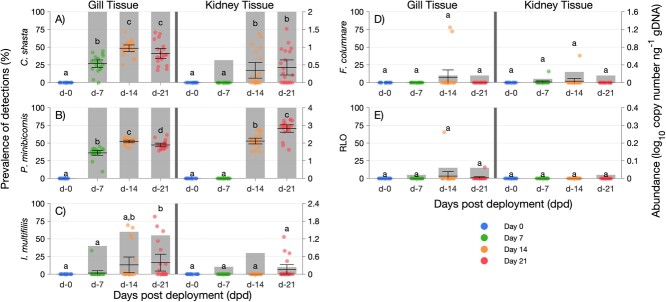
Bar plots of pathogen prevalence (bars, primary y-axis) overlaid with jitter plots of pathogen abundance (dots, secondary y-axis) in juvenile fall-run Chinook salmon at four sampling events (0,7, 14 and 21 dpd) at RBDD in gill and kidney tissues. Lowercase letters denote significantly different (*P* < 0.05) ranks, and whisker plots represent mean abundance ±2 standard error from the mean.

Prevalence and pathogen abundance varied for each pathogen and across sampling points, but both tended to increase over time. Of the eight pathogens screened, three parasites (*C. shasta*, *P. minibicornis* and *Ichthyophthirius multifiliis*) and the bacteria *Flavobacterium columnare* and Rickettsia-like organism (RLO) were detected in fish deployed at RBDD (Fig. 2). Gill and kidney were negative for all assayed pathogens in reference fish sampled at Day 0.

*Ceratonova shasta* and *P. minibicornis* were the most frequently detected pathogens, with 100% prevalence (20/20) at 7, 14 and 21 dpd for gill tissue and 14 and 21 dpd for kidney tissue. Abundance of *C. shasta* and *P. minibicornis* also increased significantly by 7 dpd when compared with 0 dpd in the gill and by 14 dpd in the kidney [Fig. 2A, B; Supplementary Table S2]. In the kidney, *P. minibicornis* continued to increase to the endpoint of the study at 21 dpd compared with 14 dpd. Detection of *C. shasta* in the intestine as measured by conventional PCR was relatively consistent with the detection in the kidney (Supplementary Fig. S1). In the intestine, positive samples were detected in 85% of samples (17/20) by 7 dpd, followed by 100% (20/20) at 14 and 21 dpd.

The additional pathogens detected (*I. multifiliis*, *F. columnare* and RLO) were often near or below 50% prevalence over the duration of the study, with pathogen abundance staying relatively stable at different time points. Prevalence of *I. multifiliis* increased above 50% by 14 and 21 dpd in gill tissue, and abundance was also significantly higher during this time compared with 0 dpd, while both prevalence and abundance in kidney tissue remained relatively low during the duration of the study (Fig. 2C). Both *F. columnare* and RLO were detected in gill and kidney by 7, 14 and 21 dpd. However, there were no differences in pathogen abundance, nor in prevalence in gill and kidney between reference fish at Day 0 and RBDD-deployed fish at 7, 14 and 21 dpd (Fig. 2D, E, respectively).

### Association between pathogen abundance and gene expression

**Figure 3:**
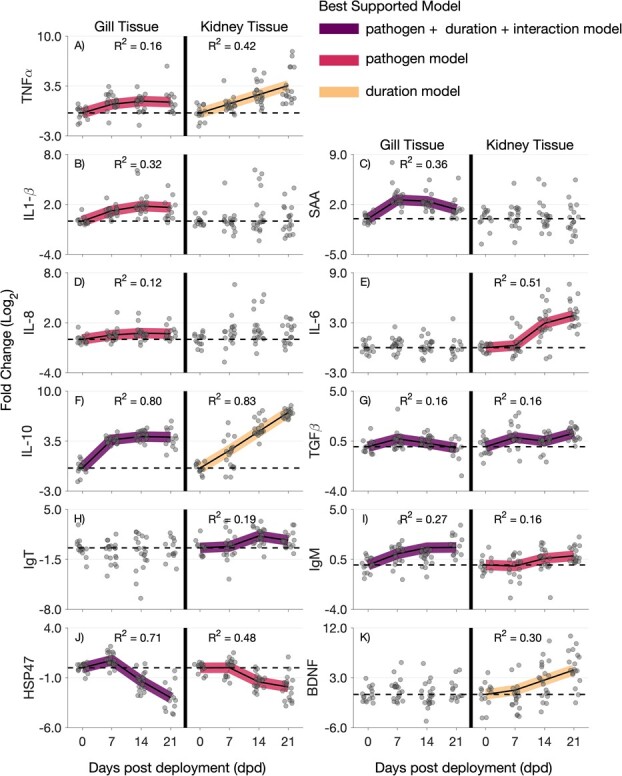
Gene expression in juvenile fall-run Chinook salmon at four sampling events (0, 7, 14 and 21 dpd) at RBDD in gill and kidney tissues presented as log_2_ fold change for the gene panel outlined in Table 2. Predictions from the best supported linear regression models (colour coded) are plotted over the gene expression data for situations where the best supported model was not the null model, with the explanatory power (*R*^2^) of a given model printed.

Results using model selection indicated that proinflammatory, anti-inflammatory, adaptive, general stress and development-related genes were associated with either pathogen abundance and/or exposure duration at varying levels over the course of the study (Fig. 3, Supplementary Tables S3–S13). For nearly all genes where the best supported model was other than the null model (i.e. where an association was found), a trend in gene upregulation was observed, except for downregulation in a stress-related gene (HSP47).

Upregulation of pro-inflammatory genes was most often associated with pathogen abundance (Model 2), and this was most often in the gills (TNFα, IL-1β, IL-8) as opposed to the kidneys (IL-6). Two additional pro-inflammatory genes were most associated with exposure duration (Model 3) in the kidney (TNFα) and with pathogen abundance and exposure duration (Model 1; global model) in the gill (SAA). Upregulation of the anti-inflammatory gene IL-10 was associated with pathogen abundance and exposure duration in the gills, but only exposure duration in the kidneys. The other anti-inflammatory gene measured (TGFβ) was associated with pathogen abundance and exposure duration in both gills and kidneys. Adaptive genes (IgT in the kidney and IgM in the gill) were associated with pathogen abundance and exposure duration, while upregulation of IgM in the kidney was associated with only pathogen abundance. A general trend of downregulation of the stress-related gene (HSP47) was associated with both pathogen abundance and exposure duration in the gill and only with pathogen abundance in the kidney. Exposure duration was associated with upregulation of the development-related gene BDNF.

The explanatory power of the regression models as assessed by *R*^2^ was highest for the association between the upregulation of the anti-inflammatory gene IL-10 in both gill and kidney tissues (i.e. *R*^2^ = 0.80 and 0.83, respectively). The model relating downregulation of the stress-related gene HSP47 in the gill and the model relating upregulation of the pro-inflammatory gene IL-6 in the kidney also had relatively high explanatory power of *R*^2^ = 0.71 and *R*^2^ = 0.51, respectively. The remainder of associations often explained much less of the variation in the data (i.e. *R*^2^ < 0.30).

### Histopathology

**Figure 4:**
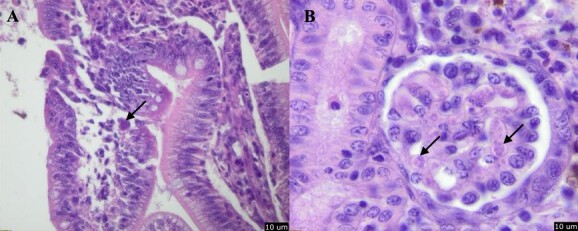
Haematoxylin and eosin-stained histological sections of juvenile fall-run Chinook salmon at 21 dpd, arrows: (A) trophozoites of *C. shasta* within lamina propria (space is an artefact of processing), (B) trophozoites of *P. minibicornis* within glomerulus with a moderate degree of glomerulonephritis. Scale bars = 10 μm.

By 21 dpd, the prevalence values of *C. shasta* in the intestine and *P. minibicornis* in kidney samples were 95% and 100%, respectively. Most (89%, 17/19) of these infections were deemed light and were accompanied by minimum to no histological signs of inflammation (rating 1). Trophozoites of *P. minibicornis* were detected in low numbers in kidney glomeruli, associated with mild glomerulonephritis (Fig. 4A) with 20% being classified as being in a diseased state (rating 2 = 4/20). Trophozoites of *C. shasta* were observed within the lamina propria and kidney granulomas in four fish, but there was no inflammation or enteronecrosis in the intestinal tissue (Fig. 4B).

## Discussion

This study characterized pathogen infections, the expression of immune, stress and development-related genes, and the association between pathogens and gene expression in sentinel juvenile fall-run Chinook salmon exposed to ambient conditions on the Sacramento River. Our findings indicate that (1) fall-run Chinook salmon are exposed to multiple pathogens when out-migrating from the Sacramento River, and (2) that gene regulation patterns were often associated with pathogen abundance. Importantly, this study observed that both 100% prevalence for two pathogens of concern and changes in gene regulation occurred at least as early as 7 dpd in gill tissues with similar trends as early as 14 dpd in kidney tissues. These gene expression findings suggest that fall-run Chinook salmon are infected with, and responding to, pathogens while out-migrating from the Sacramento River, but these also highlight that further work is needed to better connect pathogen infection risks to out-migration success.

### Pathogens

We found that sentinel Chinook salmon in the Sacramento River in spring are exposed to multiple pathogens able to cause infection and subsequent disease. Specifically, sentinel fish over a 21-day period at RBDD were primarily infected by three parasites: the myxozoans *C. shasta* and *P. minibicornis* and the ciliate *I. multifiliis*; and to a lesser extent, two bacteria: *F. columnare* and RLO. While we only discuss the three most prevalent pathogens (*C. shasta*, *P. minibicornis* and *I. multifiliis*) herein, future efforts are needed to better understand the synergistic and/or antagonistic outcomes of co-infection.

Previous studies elsewhere in the Pacific Northwest have also identified that salmon likely encounter multiple pathogens during their freshwater life stages (Jeffries *et al.*, 2014). More regionally, a study lower downstream on the Sacramento River recently reported 100% prevalence of *C. shasta* and *P. minibicornis* in gills of Chinook salmon exposed for a 14-day period (Mauduit *et al.*, 2022), and multiple years of data collection at our study site has documented frequent *C. shasta* and *P. minibicornis* infection in naturally out-migrating salmon (Foott *et al.*, 2017). Our results align with these findings and add additional information suggesting that infection may occur as early as 7 dpd. What remains unknown, however, is how these co-infections will impact the ultimate out-migration survival of Chinook salmon. Research on the dynamics between salmon and exposure to multiple pathogens at a point in time is ongoing (Sofonea *et al.*, 2015), and further investigations are needed to gain deeper insights into the interactions and consequences for salmon populations. Incorporating pathogen transmission and disease dynamics into population models of salmon survival is one approach that may improve our understanding of salmon heath in systems such as the Sacramento River and should be explored.

For *C. shasta* and *P. minibicornis*, the two most prevalent pathogens, detection in gill samples collected at 21 dpd aligned with histopathology and intestine PCR results at 21 dpd in that all had a prevalence of detections at or near 100%. It is important to note, however, that the presence of specific pathogens in fish tissue does not always imply disease establishment and progression, especially for pathogens that are ubiquitous in the environment. A combination of molecular and histopathological approaches is necessary to determine pathological significance, but still, this may not address all questions around health outcomes from exposure studies. For example, histology indicated that most fish in our study were in an infected but not diseased state. It is unknown if the infected fish we observed would have transitioned into a diseased state over time, or if they would have cleared the infection. Adding additional complexity to the interpretation of pathogen detection is that there are three genotypes (0, I, and II) of *C. shasta*, and only genotype I can cause disease in Chinook salmon (Bartholomew *et al.*, 2022). Unpublished data from our group screening environmental water samples from the Sacramento River have primarily detected genotype I by sequencing, and histology results from this study support this finding. Overall, the integration of molecular and histopathological methods, coupled with a comprehensive understanding of pathogen genotypes and their biological significance, will contribute to a more accurate understanding of disease impacts.

The third most frequently detected pathogen in this study was *I. multifiliis*, a protozoan that has been detected worldwide, but with no published detection in the Sacramento River (Dickerson, 2012; Lehman *et al.*, 2020). Throughout the Pacific Northwest, *I. multifiliis* has most often been documented during pre-spawn disease outbreaks in adult salmon (Traxler *et al.*, 1998; Belchik *et al.*, 2004). Risk factors for infection include low river discharge (Bodensteiner *et al.*, 2000) and elevated water temperature (Karvonen *et al.*, 2010). During our study period, water temperature did not rise above 17°C, and there was a large increase in river discharge from a natural precipitation event just prior to deploying fish followed by a trend in increased discharge over time associated with water management actions (Supplementary Fig. S2). Therefore, river conditions may have reduced the risk of *I. multifiliis*. However, we must also consider that the detection of *I. multifiliis* based on gill sampling used in this study was an underestimate of true prevalence, as this pathogen primarily infects the skin tissue of fish (Buchmann, 2020). Therefore, future studies should evaluate the utility of collecting swabs over the skin of salmon to detect *I. multifiliis*.

### Association between pathogen abundance and gene expression

Gene expression patterns in sentinel salmon revealed that fish exposed to ambient river conditions were responding to environmental conditions during deployment (see heat map; Supplementary Fig. S3). While this study only focused on pathogens, there are other stressors (e.g. contaminants, water temperature and even other pathogens not included in our panel) that may be associated with these expression patterns. Therefore, the findings from this work should be grounded within this context. However, our results do indicate that a portion of the gene expression observed was associated with the pathogen abundance. Furthermore, based on pathogen copy numbers alone, the majority of these associations appear to be driven by the top three pathogens (*C. shasta*, *P. minibicornis* and *I. multifiliis*) detected in this study as they made up the majority of the pathogen abundance metric used to assess the association (Supplementary Figs S4 andS5). These associations highlight the potential impact of pathogens on the physiological state of salmon and emphasize the importance of considering pathogen-related stressors when assessing the overall health of these populations.

Among the genes measuring pro-inflammatory processes, all five examined were found to be upregulated and associated with pathogen abundance in either gill or kidney tissue. In gill samples, the expression of four pro-inflammatory genes (TNFα, IL-1β, SAA and IL-8) was positively correlated with pathogen abundance, whereas in kidney samples, only one pro-inflammatory gene (IL-6) was positively correlated with pathogen abundance. This more frequent association in gill samples could be due to the gill serving as a primary entryway for many pathogens and represent immune-related cells that are produced at the pathogen entry site, controlling the ability of phagocytes to eliminate invading pathogens by driving inflammatory signals (Bose and Farnia, 1995). Of the genes associated with pathogen abundance in the gill, SAA had the highest explanatory power (*R*^2^ = 0.36). The upregulation of SAA in correlation to pathogen abundance was also observed by Mauduit *et al*. (2022) for *C. shasta* and *P. minibicornis* and may indicate that this gene is a good candidate for linking gene expression to *C. shasta* and *P. minibicornis* exposure in the Sacramento River. The only pro-inflammatory gene associated with pathogen abundance in the kidney was IL-6, which had relatively high explanatory power (*R*^2^ = 0.51). Upregulation of IL-6 was not predicted to occur until 14 dpd, which is similar to the timeline reported from gill samples in Bjork *et al.* (2014) (12 dpd) where Chinook were experimentally exposed to *C. shasta*. Why upregulation of IL-6 was not observed in gill tissues for this study is puzzling, but differences between tissue gene expression have been observed elsewhere (Hurst *et al.*, 2019). Overall, this dataset demonstrates elevation of the inflammatory response of salmon deployed in the Sacramento River over the course of the study, which may have had detrimental effects on individuals impacted by inflammation.

The strongest association between pathogen abundance and gene regulation was observed for the anti-inflammatory gene IL-10 in the gill (*R*^2^ = 0.80). Excessive IL-10 is regarded as a critical biomarker following pathogen infections in fish, where moderately controlled IL-10 expression levels indicate recovery, and overproduction is associated with severe infection (Gorgoglione *et al.*, 2013; Bailey *et al.*, 2017). In the gill, IL-10 stabilized following upregulation at 7 dpd and may therefore indicate recovery from infection. The pattern of IL-10 in the kidney, however, showed a persistent rise over the study period, but was most parsimoniously explained by the exposure duration model. Further investigation into the model selection revealed that the exposure duration model was only 1.2 times (ΔAICc = 0.38; Supplementary Table S8) more parsimonious based on evidence ratios (Burnham and Anderson, 2004) than the global model that also included the pathogen abundance term. To put this into context, the global model for gill tissue and IL-10 was over 15 000 times (ΔAICc = 19.29; Supplementary Table S8) more parsimonious than the pathogen only model. Therefore, it may be that the pattern of IL-10 in the kidney was responding to pathogen exposure, but that the exposure duration model was only slightly more parsimonious. Considering that prior work in salmonids often (Bjork *et al.*, 2014; Holzer *et al.*, 2021), but not always (Hurst *et al.*, 2019), find significant upregulation of IL-10 following infection from myxozoans and that both myxozoans were detected at 100% prevalence at 14 and 21 dpd, it seems plausible these patterns are in part due to infection.

Adaptive immune response genes (IgT and IgM) were also associated with pathogen abundance in sentinel salmon. Prior work exploring antibody response in salmon and myxozoan infection (the two main pathogens found in this study) remains unresolved but generally observed that fish susceptible to infection to have less upregulation of IgT and IgM compared with resistant fish (Barrett *et al.*, 2021; Bartholomew *et al.*, 2022). Recently however, Taggart-Murphy *et al.* (2021) unexpectedly observed that IgT and IgM were upregulated in intestinal tissue in susceptible rainbow trout as early as 7 days post-exposure and suggested that in acute high mortality cases the immune response, while mounted, may be insufficient or be too late to protect the host. As previously discussed, the Chinook salmon in our study are susceptible to the *C. shasta* genotype observed in the study system. Therefore, upregulation in gill and kidney tissues observed in this study appears to align with that by Taggart-Murphy *et al.* (2021) in that an immune response was mounted but did not protect the host as indicated by histopathological screening at 21 dpd. What remains unknown is whether the fish in our study would have cleared the infection if given more time or if the immune response would have been unsuccessful and mortality would have occurred.

Pathogen abundance was also able to describe the regulation of a heat stress gene (HSP47) in both the gill and kidney (*R*^2^ = 0.71 and 0.48, respectively). Prior research measuring heat stress and immune genes has observed both to be correlated in thermally stressed salmon, but typically in the upregulation direction (von Biela *et al.*, 2020) We observed immune genes to be upregulated out to 21 dpd, while heat stress was upregulated 7 dpd followed by downregulation 14 and 21 dpd. To put this finding into context, temperatures at RBDD were within the tolerance range of Chinook salmon (Poletto *et al.*, 2017), but fish did experience higher mean temperature at 7 dpd than at 0 dpd (Supplementary Fig. S2). Therefore, it appears there was initial heat stress in the study population at 7 dpd, but that this stress did not continue past the first week of exposure. Had temperatures continued to increase over the course of the study, we may have observed greater pathogen abundances as many pathogen–host processes are temperature dependent (Chapman *et al.*, 2021).

A significant gene expression pattern that was associated with exposure duration, but not associated with pathogen abundance, was the upregulation of the development-related gene (BDNF) in the kidney. Similar to IL-10 in the kidney, however, the exposure duration model was only 1.95 times (ΔAICc = 1.33; Supplementary Table S13) more parsimonious than the model than included pathogen abundance, and these results should be interpreted with this in mind. Expression of BDNF in kidney tissue has recently been reported to be associated with nephropathy (Tao *et al.*, 2018), and it has been further proposed as a biomarker for glomerular kidney injury (Endlich *et al.*, 2018). Considering that the model with pathogen exposure was nearly as parsimonious as the exposure duration model, the significant upregulation of BDNF in the kidney of deployed juvenile salmon could be a biomarker of kidney tissue damage, given histopathological observation of kidney tissue damage associated with *P. minibicornis* in a subset of histopathology samples.

## Conclusion

Infectious diseases will remain a conservation issue as climate change continues to reshape host–environment–agent dynamics in the Sacramento River and elsewhere (Karvonen *et al.*, 2010). To be able to predict the trajectory of these dynamics, we will require tools outlined in this study as well as others. For example, while we were able to observe an association between the immune response of salmon and pathogen abundance and tie this to histologically observed infection states, we were not able to observe the survival outcome of fish due to lethal sampling. Future work should merge non-lethal sampling methods employed in this study with observational-based approaches used to assess out-migration survival to characterize the impact of pathogen exposure more clearly on salmon population health as in Jeffries *et al.* (2014). Additionally, while our study focused on common pathogens in Chinook salmon, it did not cover all pathogens or additional stressors. Therefore, as mentioned previously, changes in gene expression could have been linked to other pathogens and/or anthropogenic factors (e.g. contaminants) necessitating cautious interpretation of the current findings. Inferences from this study were also hampered as the temporal trends of pathogen abundance represent a group-level effect and may not be accurate for individual fish as we did not follow the same fish over time. Lastly, gene expression data were normalized to reference fish sampled at 0 dpd instead of using corresponding time point controls.

The Sacramento River is a diverse system with four runs of Chinook salmon that exhibit a suite of life histories and can inhabit all parts of the river at different life stages (Fisher, 1994). Our study focused on a single run (fall run) and life stage (smolt) and found evidence that pathogens were associated with host immune function. To better understand the consequences of this pathogen–immune association across the salmon lifecycle, we recommend the implementation of a system- and life stage-wide research programme. This programme would include routine surveillance for pathogens throughout the system with sentinel and wild fish studies that would culminate in data synthesis effort that is integrated within existing fish monitoring programmes. A monitoring and data synthesis programme that builds on the methods and findings outlined here will allow for a more holistic picture of salmon conservation by improving our understanding of one of the many stressors, pathogens, affecting salmon populations, and enhance our ability to recover declining salmon populations in California.

## Supplementary Material

Web_Material_coad066

## References

[ref1] Altizer S , Bartel R, Han BA (2011) Animal migration and infectious disease risk. Science 331: 296–302. 10.1126/science.1194694. 21252339

[ref2] Atkinson SD , Hallett SL, Bartholomew JL (2018) Genotyping of individual *Ceratonova shasta* (cnidaria: Myxosporea) myxospores reveals intra-spore its-1 variation and invalidates the distinction of genotypes II and III. Parasitology 145: 1588–1593. 10.1017/S0031182018000422. 29580305

[ref3] Bailey C , Segner H, Casanova-Nakayama A, Wahli T (2017) Who needs the hotspot? The effect of temperature on the fish host immune response to *Tetracapsuloides bryosalmonae* the causative agent of proliferative kidney disease. Fish Shellfish Immunol 63: 424–437. 10.1016/j.fsi.2017.02.039. 28238860

[ref4] Barrett DE , Estensoro I, Sitjà-Bobadilla A, Bartholomew JL (2021) Intestinal transcriptomic and histologic profiling reveals tissue repair mechanisms underlying resistance to the parasite *Ceratonova shasta*. Pathogens 10: 1179. 10.3390/pathogens10091179. 34578212 PMC8467531

[ref5] Bartholomew JL , Alexander JD, Hallett SL, Alama-Bermejo G, Atkinson SD (2022) *Ceratonova shasta*: a cnidarian parasite of annelids and salmonids. Parasitology 149: 1862–1875. 10.1017/S0031182022001275. 36081219 PMC11010528

[ref6] Bass AL , Bateman AW, Connors BM, Staton BA, Rondeau EB, Mordecai GJ, Teffer AK, Kaukinen KH, Li S, Tabata AM et al. (2022) Identification of infectious agents in early marine Chinook and Coho salmon associated with cohort survival. Facets 7: 742–773. 10.1139/facets-2021-0102.

[ref7] Belchik M , Hillemeier D, Pierce RM (2004) The Klamath River Fish Kill of 2002; Analysis of Contributing Factors. Yurok Tribal Fisheries Program, 42. Yurok Tibe, Klamath, CA, pp. 2: 4

[ref8] Benjamini Y , Hochberg Y (1995) Controlling the false discovery rate: a practical and powerful approach to multiple testing. J R Stat Soc B Methodol 57: 289–300.

[ref9] von Biela VR , Bowen L, McCormick SD, Carey MP, Donnelly DS, Waters S, Regish AM, Laske SM, Brown RJ, Larson S et al. (2020) Evidence of prevalent heat stress in Yukon River Chinook salmon. Can J Fish Aquat Sci 77: 1878–1892. 10.1139/cjfas-2020-0209.

[ref10] Bjørgen H , Koppang EO (2022) Anatomy of teleost fish immune structures and organs. In K Buchmann, CJ Secombes, eds, Principles of Fish Immunology: From Cells and Molecules to Host Protection. Springer, Cham, pp. 1–30

[ref11] Bjork SJ , Bartholomew JL (2010) Invasion of *Ceratomyxa shasta* (myxozoa) and comparison of migration to the intestine between susceptible and resistant fish hosts. Int J Parasitol 40: 1087–1095. 10.1016/j.ijpara.2010.03.005. 20385137

[ref12] Bjork SJ , Zhang Y-A, Hurst CN, Alonso-Naveiro ME, Alexander JD, Sunyer JO, Bartholomew JL (2014) Defenses of susceptible and resistant Chinook salmon (*Oncorhynchus tshawytscha*) against the myxozoan parasite *Ceratomyxa shasta*. Fish Shellfish Immunol 37: 87–95. 10.1016/j.fsi.2013.12.024. 24412163 PMC3996901

[ref13] Bodensteiner LR , Sheehan RJ, Wills PS, Brandenburg AM, Lewis WM (2000) Flowing water: an effective treatment for ichthyophthiriasis. J Aquat Anim Health 12: 209–219. 10.1577/1548-8667(2000)012<0209:FWAETF>2.0.CO;2.

[ref14] Bose M , Farnia P (1995) Proinflammatory cytokines can significantly induce human mononuclear phagocytes to produce nitric oxide by a cell maturation-dependent process. Immunol Lett 48: 59–64. 10.1016/0165-2478(95)02444-1. 8847093

[ref15] Buchanan RA , Brandes PL, Skalski JR (2018) Survival of juvenile fall-run Chinook salmon through the San Joaquin River Delta, California, 2010–2015. N Am J Fish Manag 38: 663–679. 10.1002/nafm.10063.

[ref16] Buchmann K (2020) Immune response to *Ichthyophthirius multifiliis* and role of IgT. Parasite Immunol 42: 1–6. 10.1111/pim.12675. PMC7507210 31587318

[ref17] Burnham KP , Anderson DR (2004) Model Selection and Multimodel Inference. A Practical Information-Theoretic Approach, Ed 2nd. Springer New York, NY

[ref18] Burnham KP , Anderson DR, Huyvaert KP (2010) AIC model selection and multimodel inference in behavioral ecology: some background, observations, and comparisons. Behav Ecol Sociobiol 65: 23–35. 10.1007/s00265-010-1029-6.

[ref19] Chapman JM , Kelly LA, Teffer AK, Miller KM, Cooke SJ (2021) Disease ecology of wild fish: opportunities and challenges for linking infection metrics with behaviour, condition, and survival. Can J Fish Aquat Sci 78: 995–1007. 10.1139/cjfas-2020-0315.

[ref20] Connon RE , D’Abronzo LS, Hostetter NJ, Javidmehr A, Roby DD, Evans AF, Loge FJ, Werner I (2012) Transcription profiling in environmental diagnostics: health assessments in Columbia River basin steelhead (*Oncorhynchus mykiss*). Environ Sci Technol 46: 6081–6087. 10.1021/es3005128. 22587496

[ref21] Dicker RC , Coronado F, Koo D, Parrish RG (2006) Principles of Epidemiology in Public Health Practice; an Introduction to Applied Epidemiology and *Biostatistics.* Centers for Disease Control and Prevention, Atlanta, GA

[ref22] Dickerson HW (2012) Ichthyophthirius multifiliis. In PTK Woo, K Buchmann, eds, Fish Parasites: Pathobiology and Protection. CABI, Wallingford UK, pp. 55–72

[ref23] Duesund H , Nylund S, Watanabe K, Ottem KF, Nylund A (2010) Characterization of a VHS virus genotype III isolated from rainbow trout (*Oncorhynchus mykiss*) at a marine site on the west coast of Norway. Virol J 7: 2–15. 10.1186/1743-422X-7-19. 20102597 PMC2823671

[ref24] Endlich N , Lange T, Kuhn J, Klemm P, Kotb AM, Siegerist F, Kindt F, Lindenmeyer MT, Cohen CD, Kuss AW et al. (2018) BDNF: mRNA expression in urine cells of patients with chronic kidney disease and its role in kidney function. J Cell Mol Med 22: 5265–5277. 10.1111/jcmm.13762. 30133147 PMC6201371

[ref25] Evans TG , Hammill E, Kaukinen K, Schulze AD, Patterson DA, English KK, Curtis JM, Miller KM (2011) Transcriptomics of environmental acclimatization and survival in wild adult Pacific sockeye salmon (*Oncorhynchus nerka*) during spawning migration. Mol Ecol 20: 4472–4489. 10.1111/j.1365-294X.2011.05276.x. 21951593

[ref26] Fisher FW (1994) Past and present status of Central Valley Chinook salmon. Conserv Biol 8: 870–873. 10.1046/j.1523-1739.1994.08030863-5.x.

[ref27] Foott JS , Stone R, Voss S, Nichols K (2017) *Ceratonova shasta and Parvicapsula minibicornis (Phylum cnidaria: Myxosporea) Infectivity for Juvenile Chinook Salmon (Oncorhynchus tshawytscha) in the Sacramento River: August–November 2018*. In FY2016 Technical Report, California Nevada Fish Health Center, Anderson, CA.

[ref28] Gomez D , Sunyer JO, Salinas I (2013) The mucosal immune system of fish: the evolution of tolerating commensals while fighting pathogens. Fish Shellfish Immunol 35: 1729–1739. 10.1016/j.fsi.2013.09.032. 24099804 PMC3963484

[ref29] Gorgoglione B , Wang T, Secombes CJ, Holland JW (2013) Immune gene expression profiling of proliferative kidney disease in rainbow trout (*Oncorhynchus mykiss*) reveals a dominance of anti-inflammatory, antibody and t helper cell-like activities. Vet Res 44: 1–16. 10.1186/1297-9716-44-55. 23865616 PMC3733943

[ref30] Hallett SL , Bartholomew JL (2006) Application of a real-time PCR assay to detect and quantify the myxozoan parasite *Ceratomyxa shasta* in river water samples. Dis Aquat Organ 71: 109–118. 10.3354/dao071109. 16956058

[ref31] Hallett SL , Bartholomew JL (2009) Development and application of a duplex qPCR for river water samples to monitor the myxozoan parasite *Parvicapsula minibicornis*. Dis Aquat Organ 86: 39–50. 10.3354/dao02104. 19899348

[ref32] Holzer AS , Piazzon MC, Barrett D, Bartholomew JL, Sitjà-Bobadilla A (2021) To react or not to react: the dilemma of fish immune systems facing myxozoan infections. Front Immunol 12: 1–22. 10.3389/fimmu.2021.734238. PMC8481699 34603313

[ref33] Humason G (1979) Animal Tissue Techniques. William H, Freeman and Company, San Francisco, CA

[ref34] Hurst C , Alexander J, Dolan B, Jia L, Bartholomew J (2019) Outcome of within-host competition demonstrates that parasite virulence doesn't equal success in a myxozoan model system. Int J Parasitol Parasites Wildl 9: 25–35. 10.1016/j.ijppaw.2019.03.008. 30976514 PMC6441732

[ref35] Jeffries KM , Hinch SG, Gale MK, Clark TD, Lotto AG, Casselman MT, Li S, Rechisky EL, Porter AD, Welch DW et al. (2014) Immune response genes and pathogen presence predict migration survival in wild salmon smolts. Mol Ecol 23: 5803–5815. 10.1111/mec.12980. 25354752

[ref37] Johnson PT , De Roode JC, Fenton A (2015) Why infectious disease research needs community ecology. Science 349: 1259504. 10.1126/science.1259504. 26339035 PMC4863701

[ref38] Karvonen A , Rintamäki P, Jokela J, Valtonen ET (2010) Increasing water temperature and disease risks in aquatic systems: climate change increases the risk of some, but not all, diseases. Int J Parasitol 40: 1483–1488. 10.1016/j.ijpara.2010.04.015. 20580904

[ref39] Kelley GO , Zagmutt-Vergara FJ, Leutenegger CM, Adkison MA, Baxa DV, Hedrick RP (2004) Identification of a serine protease gene expressed by *Myxobolus cerebralis* during development in rainbow trout *Oncorhynchus mykiss*. Dis Aquat Organ 59: 235–248. 10.3354/dao059235. 15264720

[ref40] Kent M (2011) Infectious diseases and potential impacts on survival of Fraser River sockeye salmon. Cohen Comm Tech Rept 1: 1–58.

[ref41] Kruskal WH , Wallis WA (1952) Use of ranks in one-criterion variance analysis. J Am Stat Assoc 47: 583–621. 10.1080/01621459.1952.10483441.

[ref42] Lehman BM , Johnson RC, Adkison M, Burgess OT, Connon RE, Fangue NA, Foott JS, Hallett SL, Martinez–López B, Miller KM (2020) Disease in Central Valley Salmon: status and lessons from other systems. San Franc Estuary Watershed Sci 18: 1–31. 10.15447//sfews.2020v18iss3art2.

[ref43] Livak KJ , Schmittgen TD (2001) Analysis of relative gene expression data using real-time quantitative PCR and the 2 (−delta delta c (t)) methods. Methods 25: 402–408. 11846609 10.1006/meth.2001.1262

[ref44] Lloyd S , LaPatra S, Snekvik K, Cain K, Call D (2011) Quantitative PCR demonstrates a positive correlation between a rickettsia-like organism and severity of strawberry disease lesions in rainbow trout, *Oncorhynchus mykiss* (walbaum). J Fish Dis 34: 701–709. 10.1111/j.1365-2761.2011.01285.x. 21838713

[ref45] Mauduit F , Segarra A, Mandic M, Todgham A, Baerwald M, Schreier A, Fangue N, Connon R (2022) Understanding risks and consequences of pathogen infections on the physiological performance of outmigrating Chinook salmon. Conserv Physiol 10: coab102. 10.1093/conphys/coab102. 35492407 PMC9040276

[ref46] Miller KM , Gardner IA, Vanderstichel R, Burnley T, Angela D, Li S, Tabata A, Kaukinen KH, Ming TJ, Ginther NG (2016) Report on the Performance Evaluation of the Fluidigm Biomark Platform for High-Throughput Microbe Monitoring in Salmon. Fisheries and Oceans Canada, Ecosystems and Oceans Science. Canadian Science Advisory Secretariat, Ottawa ON

[ref47] Miller KM , Teffer A, Tucker S, Li S, Schulze AD, Trudel M, Juanes F, Tabata A, Kaukinen KH, Ginther NG et al. (2014) Infectious disease, shifting climates, and opportunistic predators: cumulative factors potentially impacting wild salmon declines. Evol Appl 7: 812–855. 10.1111/eva.12164. 25469162 PMC4227861

[ref48] Mitchell SO , Steinum TM, Toenshoff ER, Kvellestad A, Falk K, Horn M, Colquhoun DJ (2013) *Candidatus Branchiomonas cysticola* is a common agent of epitheliocysts in seawater-farmed Atlantic salmon *Salmo salar* in Norway and Ireland. Dis Aquat Organ 103: 35–43. 10.3354/dao02563. 23482383

[ref49] Myrick CA , Cech JJ (2001). Temperature effects on Chinook salmon and steelhead: a review focusing on California's Central Valley populations, Bay-Delta Modeling Forum

[ref50] Myrick CA , Cech JJ (2004) Temperature effects on juvenile anadromous salmonids in California’s Central Valley: what don’t we know? Rev Fish Biol Fish 14: 113–123. 10.1007/s11160-004-2739-5.

[ref51] Palenzuela O , Trobridge G, Bartholomew JL (1999) Development of a polymerase chain reaction diagnostic assay for *Ceratomyxa shasta*, a myxosporean parasite of salmonid fish. Dis Aquat Organ 36: 45–51. 10.3354/dao036045. 10349552

[ref52] Panangala VS , Shoemaker CA, Klesius PH (2007) Taqman real-time polymerase chain reaction assay for rapid detection of *Flavobacterium columnare*. Aquacult Res 38: 508–517. 10.1111/j.1365-2109.2007.01695.x.

[ref53] Poletto JB , Cocherell DE, Baird SE, Nguyen TX, Cabrera-Stagno V, Farrell AP, Fangue NA (2017) Unusual aerobic performance at high temperatures in juvenile Chinook salmon, *Oncorhynchus tshawytscha*. Conserv Physiol 5: cow067. 10.1093/conphys/cow067. 28078086 PMC5216678

[ref54] Rand PS (2002) Modeling feeding and growth in gulf of Alaska sockeye salmon: implications for high-seas distribution and migration. Mar Ecol Prog Ser 234: 265–280. 10.3354/meps234265.

[ref55] Ruckelshaus MH , Levin P, Johnson JB, Kareiva PM (2002) The Pacific Salmon wars: what science brings to the challenge of recovering species. Annu Rev Ecol Syst 33: 665–706. 10.1146/annurev.ecolsys.33.010802.150504.

[ref56] Sofonea MT , Alizon S, Michalakis Y (2015) From within-host interactions to epidemiological competition: a general model for multiple infections. Philos Trans R Soc B Biol Sci 370: 20140303. 10.1098/rstb.2014.0303. PMC4528501 26150669

[ref57] Sofonea MT , Alizon S, Michalakis Y (2017) Exposing the diversity of multiple infection patterns. J Theor Biol 419: 278–289. 10.1016/j.jtbi.2017.02.011. 28193485

[ref58] Taggart-Murphy L , Alama-Bermejo G, Dolan B, Takizawa F, Bartholomew J (2021) Differences in inflammatory responses of rainbow trout infected by two genotypes of the myxozoan parasite *Ceratonova shasta*. Dev Comp Immunol 114: 1–8. 10.1016/j.dci.2020.103829. PMC7655565 32846161

[ref59] Tao YS , Piao SG, Jin YS, Jin JZ, Zheng HL, Zhao HY, Lim SW, Yang CW, Li C (2018) Expression of brain-derived neurotrophic factor in kidneys from normal and cyclosporine-treated rats. BMC Nephrol 19: 1–12. 10.1186/s12882-018-0852-2. 29540150 PMC5853162

[ref60] Teffer AK , Hinch SG, Miller KM, Patterson DA, Bass AL, Cooke SJ, Farrell AP, Beacham TD, Chapman JM, Juanes F (2022) Host-pathogen-environment interactions predict survival outcomes of adult sockeye salmon (*Oncorhynchus nerka*) released from fisheries. Mol Ecol 31: 134–160. 10.1111/mec.16214. 34614262

[ref61] Teffer AK , Hinch SG, Miller KM, Patterson DA, Farrell AP, Cooke SJ, Bass AL, Szekeres P, Juanes F (2017) Capture severity, infectious disease processes and sex influence post-release mortality of sockeye salmon bycatch. Conserv Physiol 5: 1–33. 10.1093/conphys/cox017. PMC5569998 28852514

[ref62] Teffer AK , Miller KM (2019) A comparison of nonlethal and destructive methods for broad-based infectious agent screening of Chinook salmon using high-throughput qPCR. J Aquat Anim Health 31: 274–289. 10.1002/aah.10079. 31343778

[ref63] Traxler G , Richard J, McDonald T (1998) *Ichthyophthirius multifiliis* (Ich) epizootics in spawning sockeye salmon in British Columbia, Canada. J Aquat Anim Health 10: 143–151. 10.1577/1548-8667(1998)010<0143:IMIEIS>2.0.CO;2.

[ref64] Zapata A , Diez B, Cejalvo T, Gutierrez-de Frias C, Cortés A (2006) Ontogeny of the immune system of fish. Fish Shellfish Immunol 20: 126–136. 10.1016/j.fsi.2004.09.005. 15939627

