# Supplementary material for: Responses to pathogen exposure in sentinel juvenile fall-run Chinook salmon in the Sacramento River, CA
Source: Conserv Physiol. 2023 Aug 28;11(1):coad066. doi: 10.1093/conphys/coad066 (PMC10465009; doi:10.1093/conphys/coad066)
Supplement: Web_Material_coad066 [file web_material_coad066.pdf]

Supplementary Information

Title: Responses to pathogen exposure in sentinel juvenile fall-run Chinook salmon in the Sacramento River, California

Samah M. R. Abdelrazek[1], Richard E. Connon[1], Camilo Sanchez[1], Benjamin Atencio[2], Florian Mauduit[1], Brendan Lehman[2], Sascha L. Hallett[3], Stephen D. Atkinson[3], J. Scott Foott[4], Miles E. Daniels*[2]

[1] Department of Anatomy, Physiology & Cell Biology, University of California, Davis, 95616, Davis, CA, USA

[2] Institute of Marine Sciences, University of California, Santa Cruz, Affiliated with Southwest Fisheries Science Center, National Marine Fisheries Service, National Oceanic and Atmospheric Administration, 95060, Santa Cruz, CA, USA

[3] Department of Microbiology, Oregon State University, 97331, Corvallis, OR, USA

[4] California Nevada Fish Health Center, U.S. Fish and Wildlife Service, 96007, Anderson, CA, USA

*Corresponding author: University of California, Santa Cruz, 95060, Santa Cruz, CA, USA: Tel: (831) 420-3946, Email: miles.daniels@ucsc.edu

Supplementary Information Table S1: Primer sequences developed in this study and evaluated using quantitative polymerase chain reaction analyses in juvenile fall-run Chinook salmon.

Gene	Function		(F) Forward primer sequence (5′–3′)<br>(R) Reverse primer sequence (5′–3′)
Immunoglobulin T	Adaptive	F	AGC TCC GCG TGG CTA AGA AT
		R	CAG GTA CTG GGT GGT GCC AA
Heat Shock protein serpin H1	General stress	F	TCC CTT CAT CTT CCT GGT GAA
		R	CCT TTG GGT CGC ACC ATT C
Brain derived neurotropic factor	Development	F	GGC GTT GGG AAG CAG TAG AG
		R	AGC GAG GGT AAC TAA GGG GG
Glyceraldehyde-3-Phosphate Dehydrogenase	Reference gene	F	CGC GTC GCT GAC CTG TT
		R	TGT GAC ACT GGA GGG TGT GAG T

Supplementary Information Table S2: *P*-values for pathogen abundance in gill and kidney tissue of juvenile fall-run Chinook salmon at four sampling events (0, 7, 14, and 21 days post-deployment (dpd) at Red Bluff Diversion Dam. A non-parametric Kruskal-Wallis test was used to analyze differences between time points as $\log_{10}$ values. Any statistically significant results were then followed by a positive false discovery rate correction. NA represents tests where no differences between groups existed.

	Gill tissue				Kidney tissue			
C. shasta	dpd	7	14	21	dpd	7	14	21
	0	<0.001	<0.001	<0.001	0	1.00	0.003	0.002
	7	-	<0.001	0.011	7	-	0.002	<0.001
	14	-	-	0.197	14	-	-	0.752
P. minibicornis	dpd	7	14	21	dpd	7	14	21
	0	<0.001	<0.001	<0.001	0	1.00	<0.001	<0.001
	7	-	<0.001	<0.001	7	-	<0.001	<0.001
	14	-	-	0.009	14	-	-	<0.001
I. multifiliis	dpd	7	14	21	dpd	7	14	21
	0	0.483	0.054	0.002	0	NA	NA	NA
	7	-	0.054	0.003	7	-	NA	NA
	14	-	-	0.336	14	-	-	NA
F. columnare	dpd	7	14	21	dpd	7	14	21
	0	NA	NA	NA	0	NA	NA	NA
	7	-	NA	NA	7	-	NA	NA
	14	-	-	NA	14	-	-	NA
RLO	dpd	7	14	21	dpd	7	14	21
	0	NA	NA	NA	0	-	NA	NA
	7	-	NA	NA	7	-	NA	NA
	14	-	-	NA	14	-	-	NA

Supplementary Information Table S3: Model selection table for gene TNFα with regression estimates displayed for best supported model for gill (left) and kidney (right) tissues. AICc, Delta AICc, Log-Likelihood (LL), and Evidence Ratios (ER) were calculated as in Burnham *et al.*, 2010.

	Gill						Kidney					
	Model	K	AICc	Delta AICc	LL	ER	Model	K	AICc	Delta AICc	LL	ER
Model Selection	pathogen	3	217.73	0.00	-105.68	------	time	3	259.2	0.00	-126.42	------
	global	5	220.47	2.74	-104.75	3.95	global	5	262.53	3.34	-125.8	5.30
	time	3	221.9	4.17	-107.76	8.04	pathogen	3	264.34	5.14	-128.99	13.09
	null	2	228.46	10.73	-112.14	213.91	null	2	296.4	37.2	-146.11	> 1000.00

	Gill				Kidney			
	Covariate	Estimate	Std. Error	t value	Covariate	Estimate	Std. Error	t value
Regression estimates	(Intercept)	0.19	0.28	0.68	(Intercept)	-0.001	0.32	-0.007
	pathogen	0.63	0.17	3.72	time	0.17	0.024	7.17

Supplementary Information Table S4: Model selection table for gene IL-1β with regression estimates displayed for best supported model for gill (left) and kidney (right) tissues. AICc, Delta AICc, Log-Likelihood (LL), and Evidence Ratios (ER) were calculated as in Burnham *et al.*, 2010.

	Gill						Kidney					
	Model	K	AICc	Delta AICc	LL	ER	Model	K	AICc	Delta AICc	LL	ER
Model Selection	pathogen	3	203.81	0.00	-98.72	------	null	2	278.87	0.00	-137.35	------
	global	5	207.03	3.21	-98.03	4.99	pathogen	3	280.13	1.26	-136.88	1.87
	time	3	216.35	12.53	-104.99	526.72	time	3	280.52	1.64	-137.08	2.27
	null	2	229.12	25.30	-112.47	> 1000.00	global	5	282.13	3.26	-135.60	5.10

	Gill				Kidney			
	Covariate	Estimate	Std. Error	t value	Covariate	Estimate	Std. Error	t value
Regression estimates	(Intercept)	0.023	0.18	0.13				
	pathogen	0.34	0.11	3.16				

Supplementary Information Table S5: Model selection table for gene SAA with regression estimates displayed for best supported model for gill (left) and kidney (right) tissues AICc, Delta AICc, Log-Likelihood (LL), and Evidence Ratios (ER) were calculated as in Burnham *et al.*, 2010.

	Gill						Kidney					
	Model	K	AICc	Delta AICc	LL	ER	Model	K	AICc	Delta AICc	LL	ER
Model Selection	global	5	236.17	0.00	-112.60	------	null	2	286.00	0.00	-140.91	------
	pathogen	3	246.62	10.45	-120.12	186.22	pathogen	3	288.10	2.10	-140.87	2.86
	time	3	261.63	25.46	-127.63	> 1000.00	time	3	288.18	2.18	-140.91	2.98
	null	2	262.71	26.54	-129.26	> 1000.00	global	5	291.07	5.07	-140.07	12.61

	Gill				Kidney			
	Covariate	Estimate	Std. Error	t value	Covariate	Estimate	Std. Error	t value
Regression estimates	(Intercept)	0.18	0.33	0.55				
	pathogen	2.11	0.35	5.95				
	time	0.05	0.096	0.51				
	interaction	-0.10	0.047	-2.07				

Supplementary Information Table S6: Model selection table for gene IL-8 with regression estimates displayed for best supported model for gill (left) and kidney (right) tissues. AICc, Delta AICc, Log-Likelihood (LL), and Evidence Ratios (ER) were calculated as in Burnham *et al.*, 2010.

	Gill						Kidney					
	Model	K	AICc	Delta AICc	LL	ER	Model	K	AICc	Delta AICc	LL	ER
Model Selection	pathogen	3	155.50	0.00	-74.56	------	null	2	265.16	0.00	-130.49	------
	time	3	158.02	2.52	-75.82	3.53	time	3	265.53	0.37	-129.58	1.2
	global	5	160.05	4.55	-74.54	9.71	global	5	265.88	0.71	-127.47	1.43
	null	2	162.87	7.36	-79.34	39.73	pathogen	3	266.17	1.01	-129.90	1.66

	Gill				Kidney			
	Covariate	Estimate	Std. Error	t value	Covariate	Estimate	Std. Error	t value
Regression estimates	(Intercept)	0.023	0.18	0.13				
	pathogen	0.34	0.11	3.16				

Supplementary Information Table S7: Model selection table for gene IL-6 with regression estimates displayed for best supported model for gill (left) and kidney (right) tissues. AICc, Delta AICc, Log-Likelihood (LL), and Evidence Ratios (ER) were calculated as in Burnham *et al.*, 2010.

	Gill						Kidney					
	Model	K	AICc	Delta AICc	LL	ER	Model	K	AICc	Delta AICc	LL	ER
Model Selection	null	2	179.79	0.00	-87.80	------	pathogen	3	267.20	0.00	-130.42	------
	pathogen	3	181.84	2.05	-87.73	2.79	global	5	269.21	2.01	-129.14	2.73
	time	3	181.97	2.18	-87.80	2.98	time	3	269.63	2.42	-131.63	3.36
	global	5	186.00	6.21	-87.51	22.28	null	2	316.5	49.29	-156.16	> 1000.00

	Gill				Kidney			
	Covariate	Estimate	Std. Error	t value	Covariate	Estimate	Std. Error	t value
Regression estimates					(Intercept)	0.26	0.27	0.97
					pathogen	1.25	0.15	8.60

Supplementary Information Table S8: Model selection table for gene IL-10 with regression estimates displayed for best supported model for gill (left) and kidney (right) tissues. AICc, Delta AICc, Log-Likelihood (LL), and Evidence Ratios (ER) were calculated as in Burnham *et al.*, 2010.

	Gill						Kidney					
	Model	K	AICc	Delta AICc	LL	ER	Model	K	AICc	Delta AICc	LL	ER
Model Selection	global	5	173.53	0.00	-81.28	------	time	3	231.10	0.00	-112.37	------
	pathogen	3	192.82	19.29	-93.22	> 1000.00	global	5	231.48	0.38	-110.27	1.21
	time	3	232.62	59.10	-113.12	> 1000.00	pathogen	3	257.36	26.26	-125.50	> 1000.00
	null	2	278.31	104.78	-137.06	> 1000.00	null	2	353.06	121.96	-174.44	> 1000.00

	Gill				Kidney			
	Covariate	Estimate	Std. Error	t value	Covariate	Estimate	Std. Error	t value
Regression estimates	(Intercept)	0.040	0.21	0.19	(Intercept)	-0.20	0.26	-0.73
	pathogen	2.15	0.22	9.64	time	0.35	0.02	18.24
	time	0.29	0.06	4.80				
	interaction	-0.15	0.03	-5.19				

Supplementary Information Table S9: Model selection table for gene TGFβ with regression estimates displayed for best supported model for gill (left) and kidney (right) tissues. AICc, Delta AICc, Log-Likelihood (LL), and Evidence Ratios (ER) were calculated as in Burnham *et al.*, 2010.

	Gill						Kidney					
	Model	K	AICc	Delta AICc	LL	ER	Model	K	AICc	Delta AICc	LL	ER
Model Selection	global	5	151.30	0.00	-70.17	------	global	5	181.46	0.00	-85.26	------
	null	2	159.68	8.37	-77.75	65.73	time	3	184.02	2.55	-88.83	3.59
	pathogen	3	160.72	9.42	-77.17	110.91	pathogen	3	189.45	7.99	-91.55	54.37
	time	3	161.40	10.10	-77.51	155.66	null	2	190.11	8.65	-92.97	75.55

	Gill				Kidney			
	Covariate	Estimate	Std. Error	t value	Covariate	Estimate	Std. Error	t value
Regression estimates	(Intercept)	-0.05	0.18	-0.25	(Intercept)	0.13	0.21	0.63
	pathogen	0.70	0.19	3.70	pathogen	-0.76	0.32	-2.35
	time	0.05	0.05	0.89	time	0.10	0.04	2.79
	interaction	-0.06	0.03	-2.33	interaction	0.02	0.02	1.12

Supplementary Information Table S10: Model selection table for gene IgT with regression estimates displayed for best supported model for gill (left) and kidney (right) tissues. AICc, Delta AICc, Log-Likelihood (LL), and Evidence Ratios (ER) were calculated as in Burnham *et al.*, 2010.

	Gill						Kidney					
	Model	K	AICc	Delta AICc	LL	ER	Model	K	AICc	Delta AICc	LL	ER
Model Selection	null	2	263.49	0.00	-129.65	------	global	5	234.51	0.00	-111.79	------
	pathogen	3	264.61	1.12	-129.12	1.75	pathogen	3	235.70	1.19	-114.67	1.81
	time	3	265.50	2.01	-129.56	2.74	time	3	238.58	4.07	-116.11	7.64
	global	5	267.52	4.03	-128.28	7.52	null	2	245.35	10.84	-120.59	225.57

	Gill				Kidney			
	Covariate	Estimate	Std. Error	t value	Covariate	Estimate	Std. Error	t value
Regression estimates					(Intercept)	-0.02	0.31	-0.05
					pathogen	1.38	0.47	2.95
					time	0.03	0.05	0.54
					interaction	-0.06	0.03	-2.34

Supplementary Information Table S11: Model selection table for gene IgM with regression estimates displayed for best supported model for gill (left) and kidney (right) tissues. AICc, Delta AICc, Log-Likelihood (LL), and Evidence Ratios (ER) were calculated as in Burnham *et al.*, 2010.

	Gill						Kidney					
	Model	K	AICc	Delta AICc	LL	ER	Model	K	AICc	Delta AICc	LL	ER
Model Selection	global	5	183.89	0.00	-86.46	------	pathogen	3	193.92	0.00	-93.78	------
	time	3	186.65	2.76	-90.14	3.98	time	3	195.26	1.35	-94.45	1.96
	pathogen	3	190.99	7.09	-92.31	34.7	global	5	198.12	4.20	-93.59	8.17
	null	2	201.82	17.92	-98.82	> 1000.00	null	2	204.67	10.76	-100.25	216.93

	Gill				Kidney			
	Covariate	Estimate	Std. Error	t value	Covariate	Estimate	Std. Error	t value
Regression estimates	(Intercept)	-0.04	0.23	-0.17	(Intercept)	-0.10	0.16	-0.60
	pathogen	0.28	0.24	1.16	pathogen	0.32	0.09	3.72
	time	0.22	0.07	3.34				
	interaction	-0.09	0.0	-2.67				

Supplementary Information Table S12: Model selection table for gene HSP47 with regression estimates displayed for best supported model for gill (left) and kidney (right) tissues. AICc, Delta AICc, Log-Likelihood (LL), and Evidence Ratios (ER) were calculated as in Burnham *et al.*, 2010.

	Gill						Kidney					
	Model	K	AICc	Delta AICc	LL	ER	Model	K	AICc	Delta AICc	LL	ER
Model Selection	global	5	182.38	0.00	-85.71	------	pathogen	3	189.93	0.00	-91.78	------
	time	3	216.82	34.44	-105.22	> 1000.00	global	5	193.84	3.91	-91.45	7.05
	pathogen	3	251.53	69.15	-122.58	> 1000.00	time	3	195.57	5.64	-94.60	16.78
	null	2	263.71	81.33	-129.76	> 1000.00	null	2	234.26	44.33	-115.04	> 1000.00

	Gill				Kidney			
	Covariate	Estimate	Std. Error	t value	Covariate	Estimate	Std. Error	t value
Regression estimates	(Intercept)	0.05	0.23	0.21	(Intercept)	0.01	0.16	0.07
	pathogen	1.37	0.24	5.74	pathogen	-0.67	0.08	-8.01
	time	0.07	0.07	1.12				
	interaction	-0.18	0.03	-5.56				

Supplementary Information Table S13: Model selection table for gene BDNF with regression estimates displayed for best supported model for gill (left) and kidney (right) tissues. AICc, Delta AICc, Log-Likelihood (LL), and Evidence Ratios (ER) were calculated as in Burnham *et al.*, 2010.

	Gill						Kidney					
	Model	K	AICc	Delta AICc	LL	ER	Model	K	AICc	Delta AICc	LL	ER
Model Selection	null	2	283.33	0.00	-139.57	------	time	3	354.89	0.00	-174.26	------
	time	3	285.41	2.08	-139.52	2.84	pathogen	3	356.22	1.33	-174.93	1.95
	pathogen	3	285.51	2.18	-139.57	2.98	global	5	358.00	3.11	-173.53	4.74
	global	5	287.34	4.01	-138.19	7.43	null	2	378.80	23.91	-187.31	> 1000.00

	Gill				Kidney			
	Covariate	Estimate	Std. Error	t value	Covariate	Estimate	Std. Error	t value
Regression estimates					(Intercept)	-1.06	0.63	-1.67
					Time	0.26	0.05	5.54

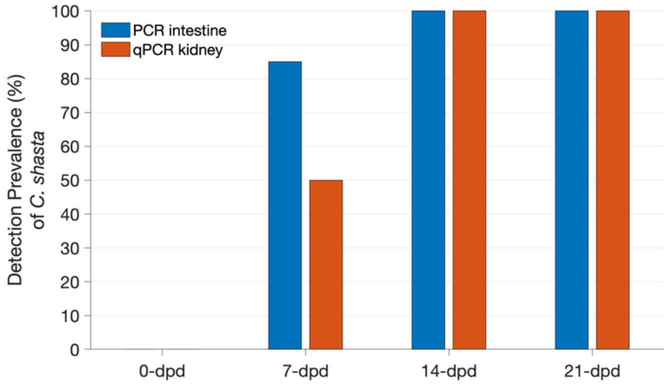

Supplementary Information Figure S1: Comparison of *Ceratonova shasta* detection prevalence in intestine (via PCR) and kidney (via qPCR) in juvenile fall-run Chinook salmon at four sampling events (0, 7, 14, and 21 days post-deployment (dpd)) at Red Bluff Diversion Dam, Sacramento River, California.

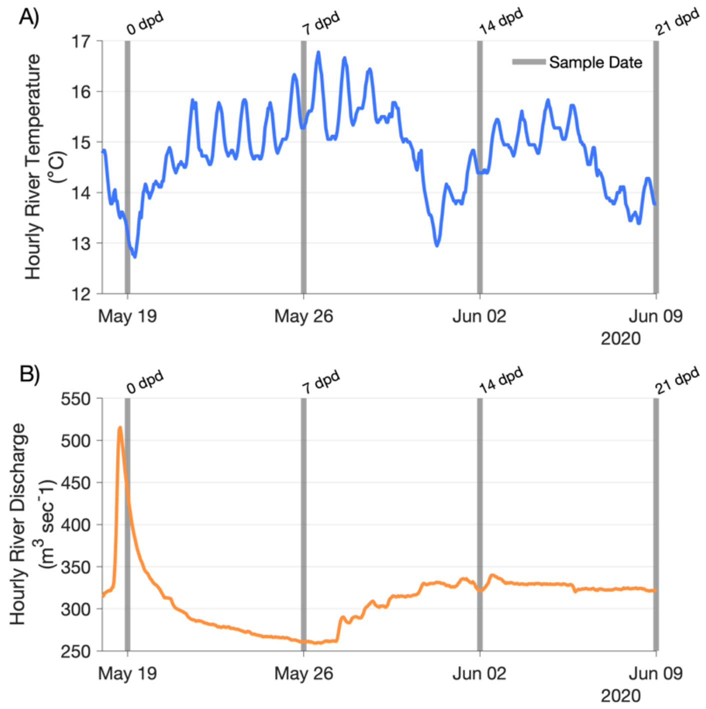

Supplementary Information Figure S2: **A)** Water temperature (°C) and **B)** river discharge (m^3 sec^{-1}), measured during the 21-day study at the RBDD. Data were collected every 15 minutes at the RBD monitoring station (cdec.water.ca.gov). Vertical grey lines represent the sample dates. Note that fish at the SWFSC were held at a temperature of 13.6 °C.

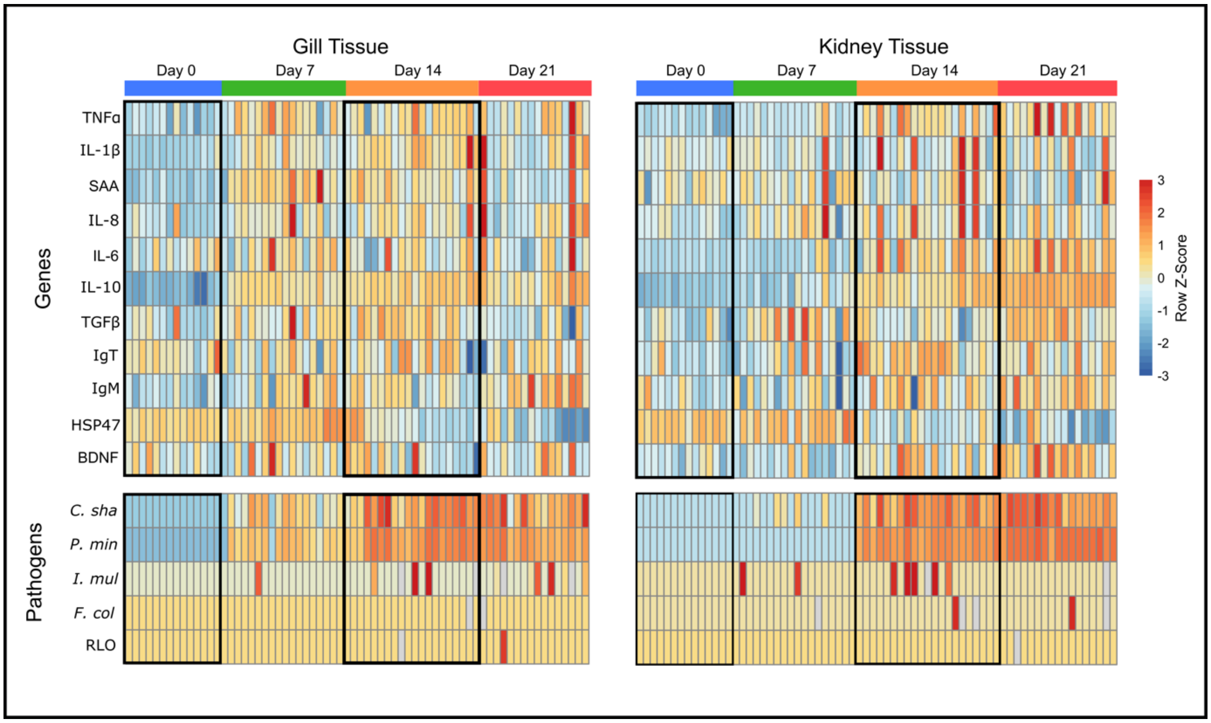

Supplementary Information Figure S3: Heat map depicting gene expression and pathogen detection in gill (left panel) and kidney (right panel), in juvenile fall-run Chinook salmon at four sampling events (0, 7, 14, and 21 days post-deployment) at Red Bluff Diversion Dam, Sacramento River, California. Data are presented as Z-scores [not calibrated to controls] to aid visual clarity across measurements with different units.

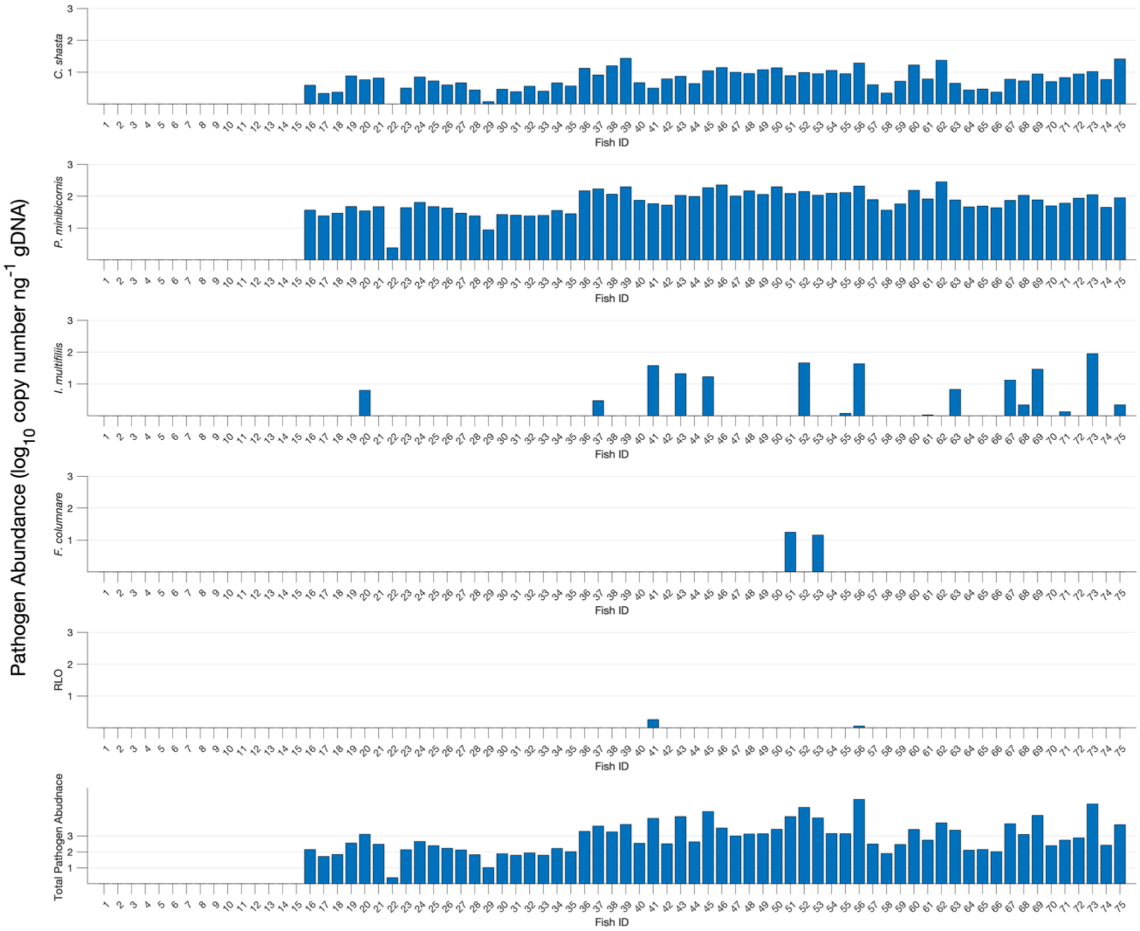

Supplementary Information Figure S4: Comparison of individual levels of pathogen abundance detected in gills for a given study fish for the five pathogens detected compared to the total pathogen abundance used as the metric for assessing the association between pathogen and gene expression.

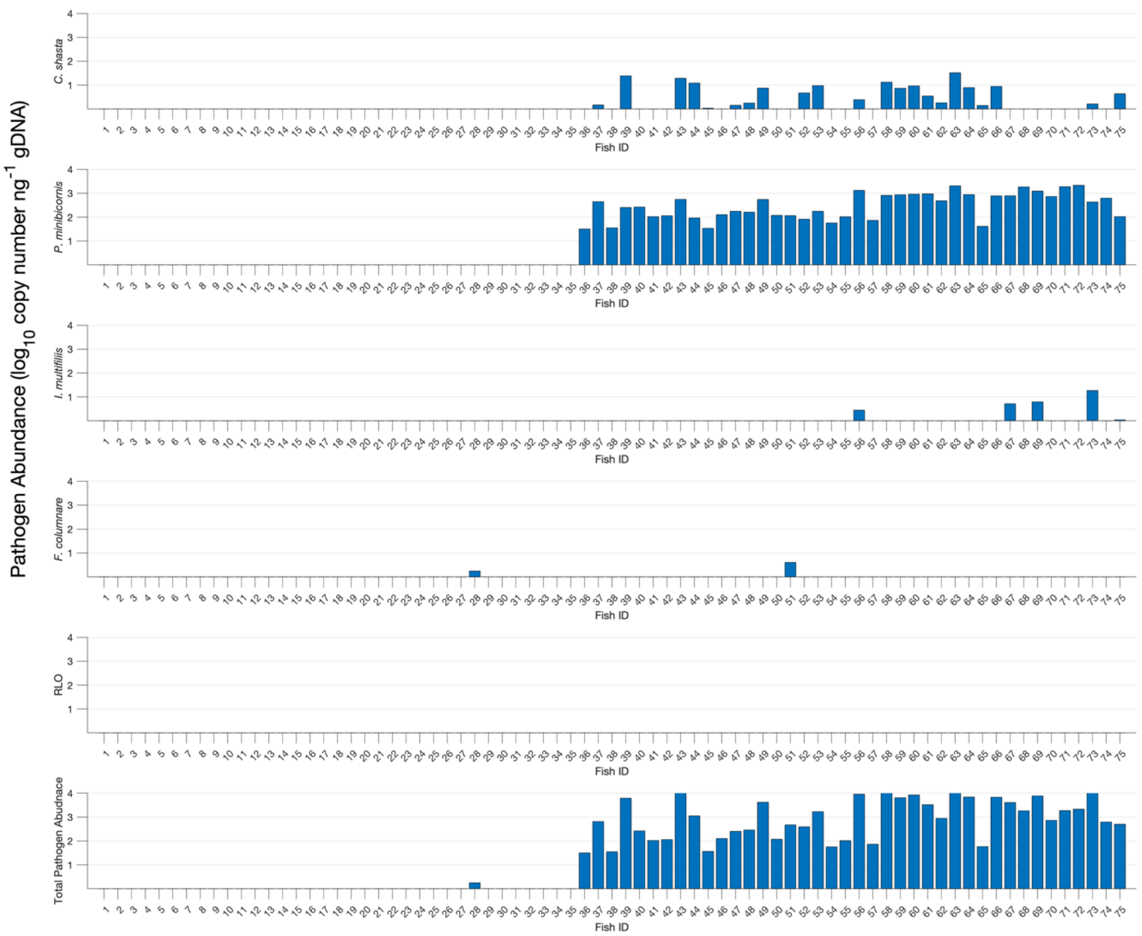

Supplementary Information Figure S5: Comparison of individual levels of pathogen abundance detected in kidneys for a given study fish for the five pathogens detected compared to the total pathogen abundance used as the metric for assessing the association between pathogen and gene expression.
